# Evaluation of 2D super-resolution ultrasound imaging of the rat renal vasculature using ex vivo micro-computed tomography

**DOI:** 10.1038/s41598-021-03726-6

**Published:** 2021-12-21

**Authors:** Sofie Bech Andersen, Iman Taghavi, Hans Martin Kjer, Stinne Byrholdt Søgaard, Carsten Gundlach, Vedrana Andersen Dahl, Michael Bachmann Nielsen, Anders Bjorholm Dahl, Jørgen Arendt Jensen, Charlotte Mehlin Sørensen

**Affiliations:** 1 Department of Biomedical Sciences, University of Copenhagen, 2200 Copenhagen, Denmark; 2 Department of Radiology, Rigshospitalet, 2100 Copenhagen, Denmark; 3 Center for Fast Ultrasound Imaging, Department of Health Technology, Technical University of Denmark, 2800 Lyngby, Denmark; 4 Department of Applied Mathematics and Computer Science, Technical University of Denmark, 2800 Lyngby, Denmark; 5 Department of Physics, Technical University of Denmark, 2800 Lyngby, Denmark; 6 Department of Clinical Medicine, University of Copenhagen, 2200 Copenhagen, Denmark

**Keywords:** Kidney, Preclinical research, Imaging techniques

## Abstract

Super-resolution ultrasound imaging (SRUS) enables in vivo microvascular imaging of deeper-lying tissues and organs, such as the kidneys or liver. The technique allows new insights into microvascular anatomy and physiology and the development of disease-related microvascular abnormalities. However, the microvascular anatomy is intricate and challenging to depict with the currently available imaging techniques, and validation of the microvascular structures of deeper-lying organs obtained with SRUS remains difficult. Our study aimed to directly compare the vascular anatomy in two in vivo 2D SRUS images of a Sprague–Dawley rat kidney with ex vivo *μ*CT of the same kidney. Co-registering the SRUS images to the *μ*CT volume revealed visually very similar vascular features of vessels ranging from ~ 100 to 1300 μm in diameter and illustrated a high level of vessel branching complexity captured in the 2D SRUS images. Additionally, it was shown that it is difficult to use *μ*CT data of a whole rat kidney specimen to validate the super-resolution capability of our ultrasound scans, i.e*.*, validating the actual microvasculature of the rat kidney. Lastly, by comparing the two imaging modalities, fundamental challenges for 2D SRUS were demonstrated, including the complexity of projecting a 3D vessel network into 2D. These challenges should be considered when interpreting clinical or preclinical SRUS data in future studies.

## Introduction

Super-resolution ultrasound imaging (SRUS) enables in vivo investigation of the microvasculature at clinically relevant depths^1–6^. The technique depicts the vascular architecture at levels below 100 μm, magnitudes lower than the currently available clinical imaging modalities for in-depth imaging, such as CT and MRI. In vivo imaging of the microvasculature at these levels opens new possibilities for investigating disease development and treatment efficacy in, e.g*.*, diabetes or cancer^7–10^.

The fundamental principle of SRUS is localization and tracking of single intravascular microbubbles (MBs). The final SRUS image is an accumulation of thousands of MB trajectories, each representing one MB’s most likely route through the vasculature. More accurate localization and tracking are currently achieved when the MBs are spatially separated in each image frame. Accordingly, dense and well-perfused vascular networks such as the renal vasculature challenge MB tracking, especially since reasonable data acquisition time is critical^11–14^. In addition, a single vessel is represented by a given number of MB trajectories, making the vessel diameter dependent on the intraluminal distribution and number of MBs that pass through. Lastly, capturing a complex 3D vascular network in 2D is challenging due to overlaying vessels and vessels that traverse the elevational plane. Since the localization and tracking of MBs are subject to uncertainty, it is central to compare the structures depicted in the SRUS images with other modalities. Microphantoms have been used to validate the spatial accuracy and precision of the MB localization^15,16^. Yet, the phantoms do not reproduce the conditions from in vivo SRUS, where the complex vascular structures, variations in blood flow, and tissue motion complicate SRUS image formation. Studies on cancer models in chick embryos and mice have correlated SRUS results to histological measurements of microvessel density or vessel area fraction^9,10^. However, it is difficult to co-register the thin histological sections and the SRUS images for comparison of the exact same areas. One of the first studies with SRUS compared an SRUS image of a thin mouse ear with an optical image of the same vessels^17^. The chorioallantoic membrane of ex ovo chicken embryos has also been used as an in vivo ‘phantom’ for validation of SRUS; like the mouse ear, the anatomy of the membrane’s thin-layered vascular bed allows co-registration of the SRUS images with optical imaging of the same field-of-view^18–20^. These approaches have coupled SRUS images directly with accurate images of the microvascular anatomy. The optical imaging techniques are restricted to superficial structures, and can also be limited in providing a ground truth, e.g*.*, due to image contrast. For deeper-lying organs, other approaches are necessary to investigate the accuracy of the vascular anatomy in the SRUS images. The vessel fraction area from SRUS images has been correlated with measures of relative blood volume from ex vivo *μ*CT of mice tumors^10^. In rabbit lymph nodes, the distribution of vessel diameters measured with *μ*CT and SRUS were in good agreement, with a peak diameter between 10 and 20 μm^21^. However, in these two studies, the imaging modalities were not directly co-registered and compared. Another study compared SRUS images of the vasa vasorum around rabbit femoral arteries with ex vivo *μ*CT of the same area and found corresponding results^22^. Lastly, CT angiography of larger arteries (mostly mm-sized) in the human brain was used for comparison with corresponding transcranial SRUS images^23^.

Ex vivo *μ*CT can also produce detailed images of the dense renal microvasculature in rodents^24–29^. Therefore, our study aimed to directly compare the vascular anatomy shown in two in vivo 2D SRUS images of a Sprague–Dawley rat kidney with ex vivo *μ*CT of the same kidney. For this comparison, we aimed at estimating the proportion of vessels in the SRUS images that were also captured in the *μ*CT. We expected the large vessels to be resolved in both modalities and that the discrepancy between resolved vessels became higher with smaller vessel diameters. Reaching these goals required imaging the same areas of the same rat kidney with both in vivo SRUS and ex vivo *μ*CT, co-registering the two modalities, segmenting the vessels in each of the modalities, and quantifying the overlap of the two segmentations.

## Results

### Image co-registration and visual comparison

**Figure 1:**
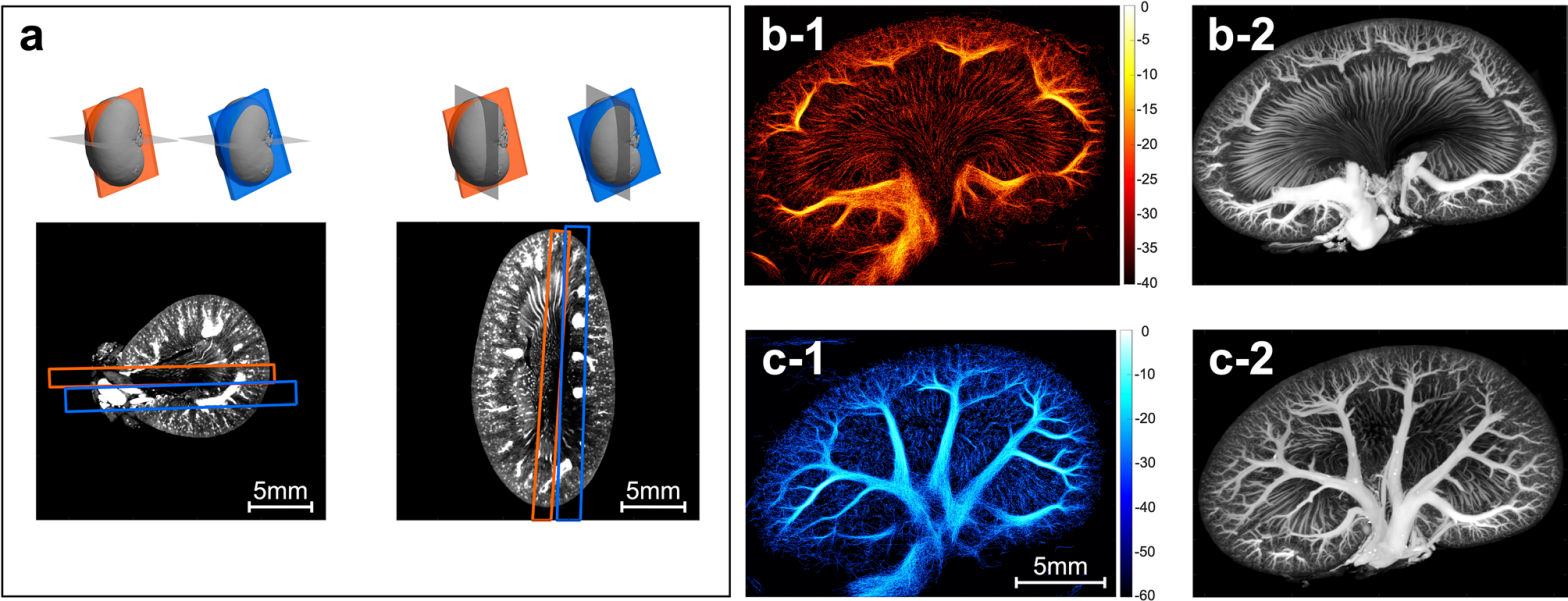
Co-registration of *μ*CT and super-resolution ultrasound images. (**a**) Co-registration of the two 2D super-resolution ultrasound images to the *μ*CT volume. The images show the two ultrasound fields of view in the *μ*CT coordinate system in an axial *μ*CT slice to the left and a sagittal *μ*CT slice to the right. (**b-1**,**c-1**) show the two super-resolution ultrasound images (intensity maps). The images are log-scaled with a dynamic range of 40 and 60 dB, respectively. The color bar shows the value of intensity after logarithmic compression. Intensity corresponds to the number of detected microbubbles. (**b-2**,**c-2**) show the *μ*CT maximum intensity projections in the super-resolution ultrasound image overlap.

**Figure 2:**
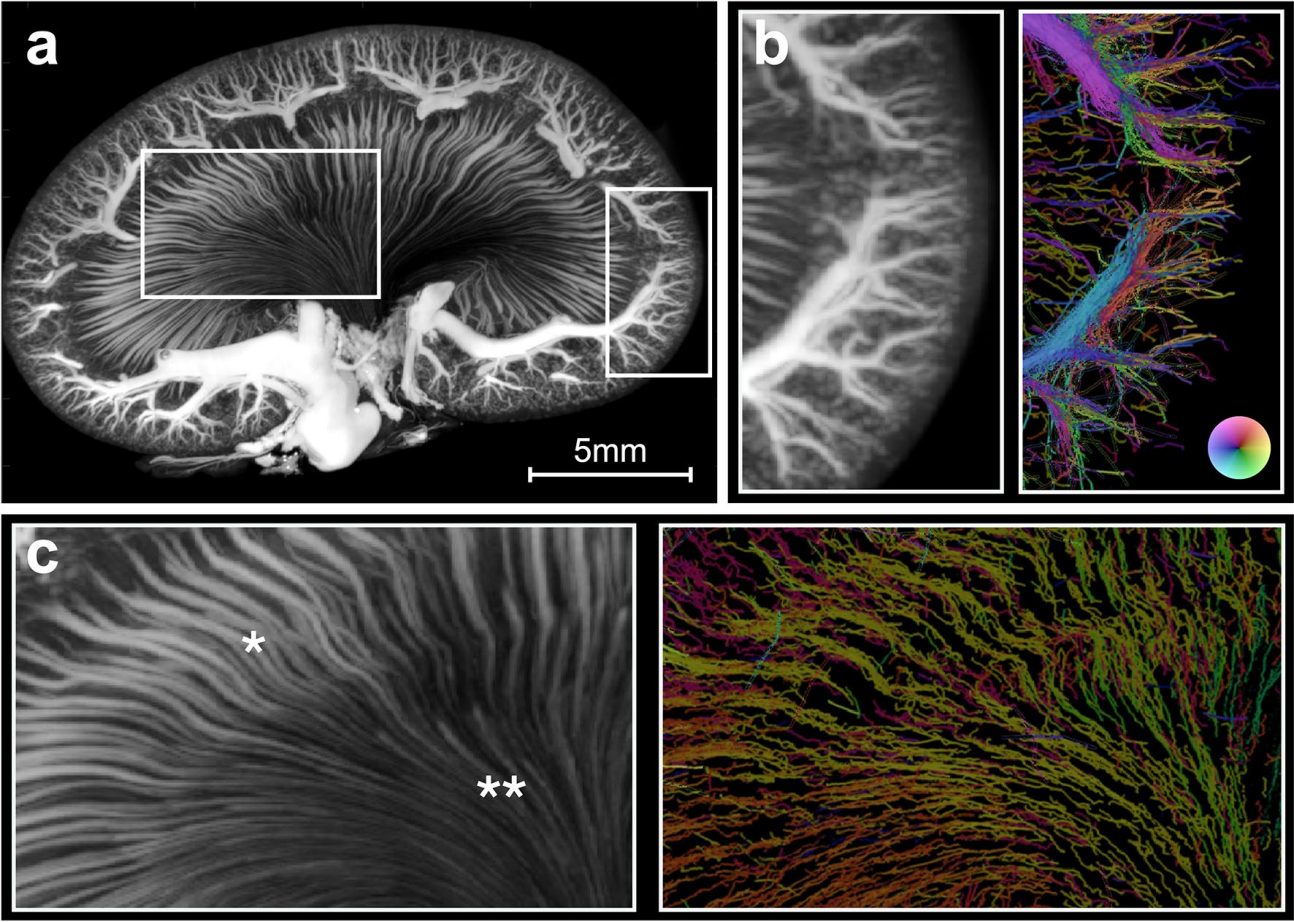
Visual comparison of the *μ*CT and microbubble track map. (**a**) *μ*CT maximum intensity projection in the super-resolution ultrasound image overlap of scan 1. The two marked regions are up-scaled and compared with the corresponding unfiltered microbubble track map in (**b**) and (**c**) (for visualization, the color transparency in the microbubble track map is scaled by the intensity map). (**b**) Shows a part of the renal cortex. (**c**) Shows a part of the renal medulla with the vasa recta. Notice how the color wheel in the microbubble track map allows separation of arteries and veins according to flow direction, e.g*.*, in (**c**) with the descending (orange/yellow/green) and ascending (purple/blue) vasa recta of the medulla. * marks outer medulla, ** marks inner medulla.

Maximum intensity projections (MIPs) of the *μ*CT slices approximately covering the volume depicted in the two SRUS images were created by co-registering the SRUS images to the *μ*CT volume. In Fig. 1a, the two ultrasound fields of view are shown in the *μ*CT coordinate system. Figure 1b-1 shows an SRUS image acquired down the center of the kidney (scan 1) to display both the cortical vasculature and the vasa recta of the medulla, and Fig. 1c-1 shows a scan obtained more ventrally (scan 2), showing the segmental and arcuate vessels and their branches. The corresponding *μ*CT MIPs are shown in Fig. 1b-2,c-2.

Very similar vascular features were evident from visual inspection of the corresponding images from the two imaging modalities. In Fig. 2, some of the similarities are shown in up-scaled examples from the *μ*CT MIP of scan 1 compared with the corresponding MB track map composed of the MB trajectories, where the color of the trajectories indicates the MB flow direction. For example, both imaging modalities clearly displayed the wavy course of the outer medulla vasa recta and the straighter course of the inner medulla vasa recta (Fig. 2c). An advantageous feature of the MB track maps is the immediate distinction between vessels with arterial and venous flow, e.g*.*, the descending and ascending vasa recta: a feature not immediately extractable from the *μ*CT.

### Quantification of similarities between super-resolution ultrasound images and ex vivo μCT

**Figure 3:**
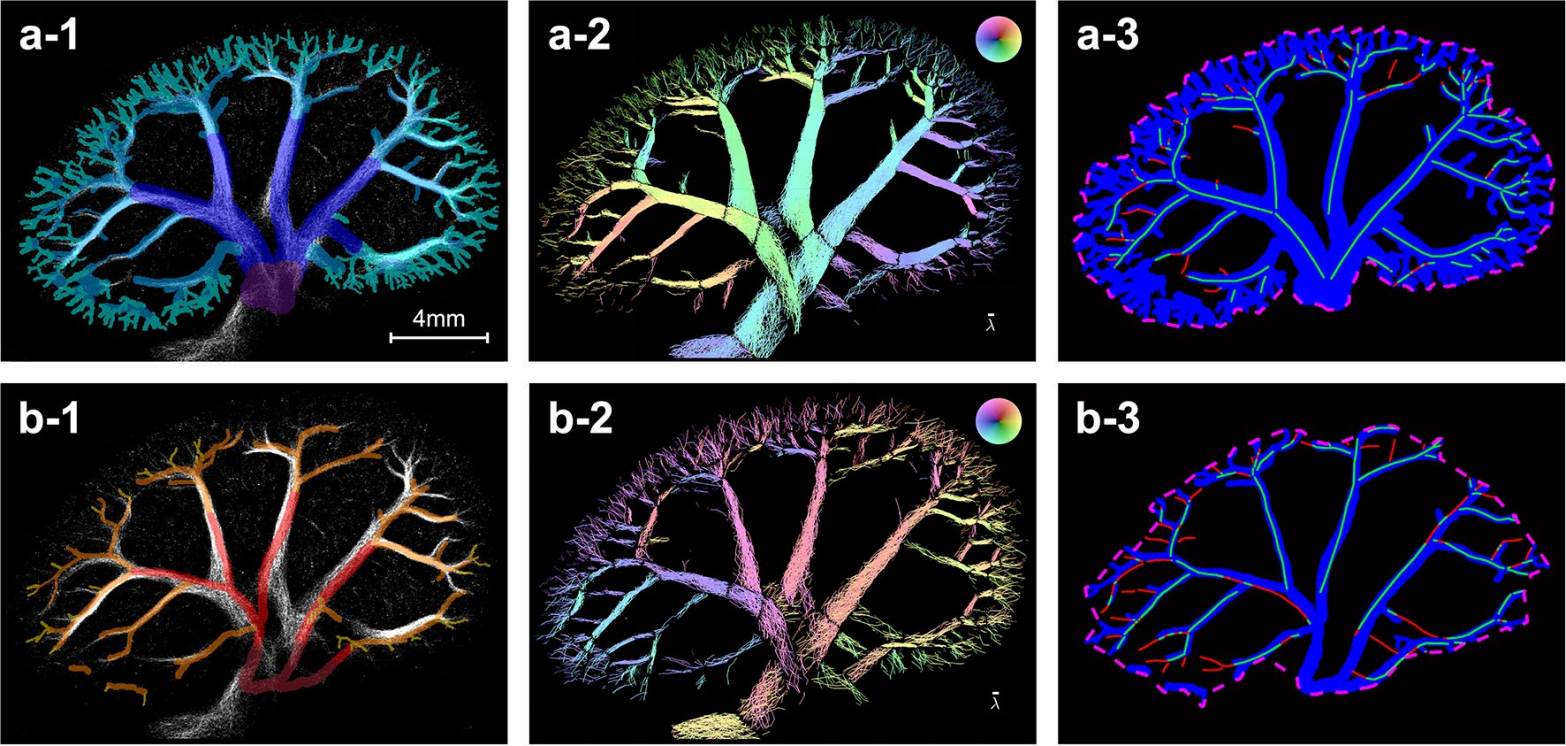
Vessel overlap estimation. (**a**) Veins, (**b**) Arteries. (**a-1**,**b-1**) Manually drawn veins and arteries on the *μ*CT in the super-resolution ultrasound scan 2 overlap (the colored *μ*CT segmentation is displayed on top of a white super-resolution ultrasound image). Purple = renal vein branches, dark blue = segmental veins, blue = arcuate veins, turquoise = cortical radial veins, dark red = renal artery branches, red = segmental arteries, orange = arcuate arteries, yellow = cortical radial arteries. (**a-2,b-2**) Filtered microbubble track maps including only regions labeled as “segmental or larger arcuate vessels” or “cortex” and separated into a map with only vein (**a-2**) and only artery (**b-2**) tracks, respectively. (**a-3**,**b-3**) Overlap of the manually drawn vessel centerlines in the super-resolution image (green and red lines) and *μ*CT vessel ROIs (blue) inside the *μ*CT ROI region (pink dashed line). The green centerlines are overlapping the *μ*CT ROIs while the red centerlines are not.

The proportion of vessels in the SRUS scan 2 that were also captured in the *μ*CT was assessed by finding the percentage of vessel overlap after vessel segmentation. On the *μ*CT, the vessel centerlines of the visible, contrast-filled segmental, arcuate, and cortical radial arteries and veins were manually drawn. The majority of the vessels resolved for vessel centerline segmentation in the *μ*CT were veins (621 vein segments, size range: ~ 80–1400 μm). Due to the smaller diameter of the arteries, mainly the segmental and arcuate arteries were visible, but even some arcuate arteries had such a small diameter that they were indistinguishable from nearby structures (110 artery segments, size range: ~ 50–500 μm). The *μ*CT centerlines were projected into the coordinate system of the 2D SRUS images, and to cover the contrast-filled area, the centerlines were dilated based on approximate expected vessel diameters extracted from examples of diameter measurements of the segmental, arcuate, and cortical radial arteries and veins on the *μ*CT. This dilation was used only to create two regions of interest (ROIs) for vessel overlap estimation: one for all the vein segments (Fig. 3a-1) and one for all the artery segments (Fig. 3b-1). Dilated volumes were not used for further quantification, so possible biases due to the choice of dilation did not propagate to the rest of the analysis. In these ROIs, we could reasonably expect to observe MB tracks. The MB track map from scan 2 was filtered to include only regions labeled as “segmental or larger arcuate vessels” or “cortex” (see the “Materials and methods” section for clarification). Based on arterial flow direction determination during MB track map filtering, the image was separated into an image displaying only the vein tracks (Fig. 3a-2) and one displaying only the artery tracks (Fig. 3b-2), respectively. Then, the vessel centerlines of well-defined MB trajectories in the filtered MB track map were manually plotted. Of the SRUS vein centerlines, 85% were recovered within the *μ*CT ROI, while only 65% of the SRUS artery centerlines were recovered (Fig. 3a-3,b-3). Even though the percentage of artery overlap was lower, many of the SRUS artery centerlines had a corresponding *μ*CT artery ROI in close proximity, as evident from Fig. 3b-3. This illustrates how the discrepancy in vessel overlap becomes higher with smaller vessel diameters.

The percentage of all the MB tracks (all non-zero pixels in the filtered MB track map) covered by the *μ*CT ROIs was similar: 77% of the vein MB tracks and 44% of the artery MB tracks were recovered inside the respective *μ*CT ROIs (Supplementary Fig. S1). As this comparison included more of the smaller arcuate and cortical radial vessels than the centerline comparison, a lower overlap percentage was expected. The reported overlap percentages are difficult to put into context. The vessel structures of the kidney are very densely organized, and some level of spurious overlap can always be expected. As a control, we mirrored the *μ*CT ROIs along the central axial axis, which resulted in a drop in the overlap percentages to 50% (veins) and 24% (arteries) for the SRUS vessel centerlines and 51% (veins) and 23% (arteries) for the MB tracks (Supplementary Fig. S2). This procedure was considered likely to represent a best-case scenario for spurious overlap, and the percentages would most likely be similar or even smaller for a random match of the SRUS images and *μ*CT volume.

**Figure 4:**
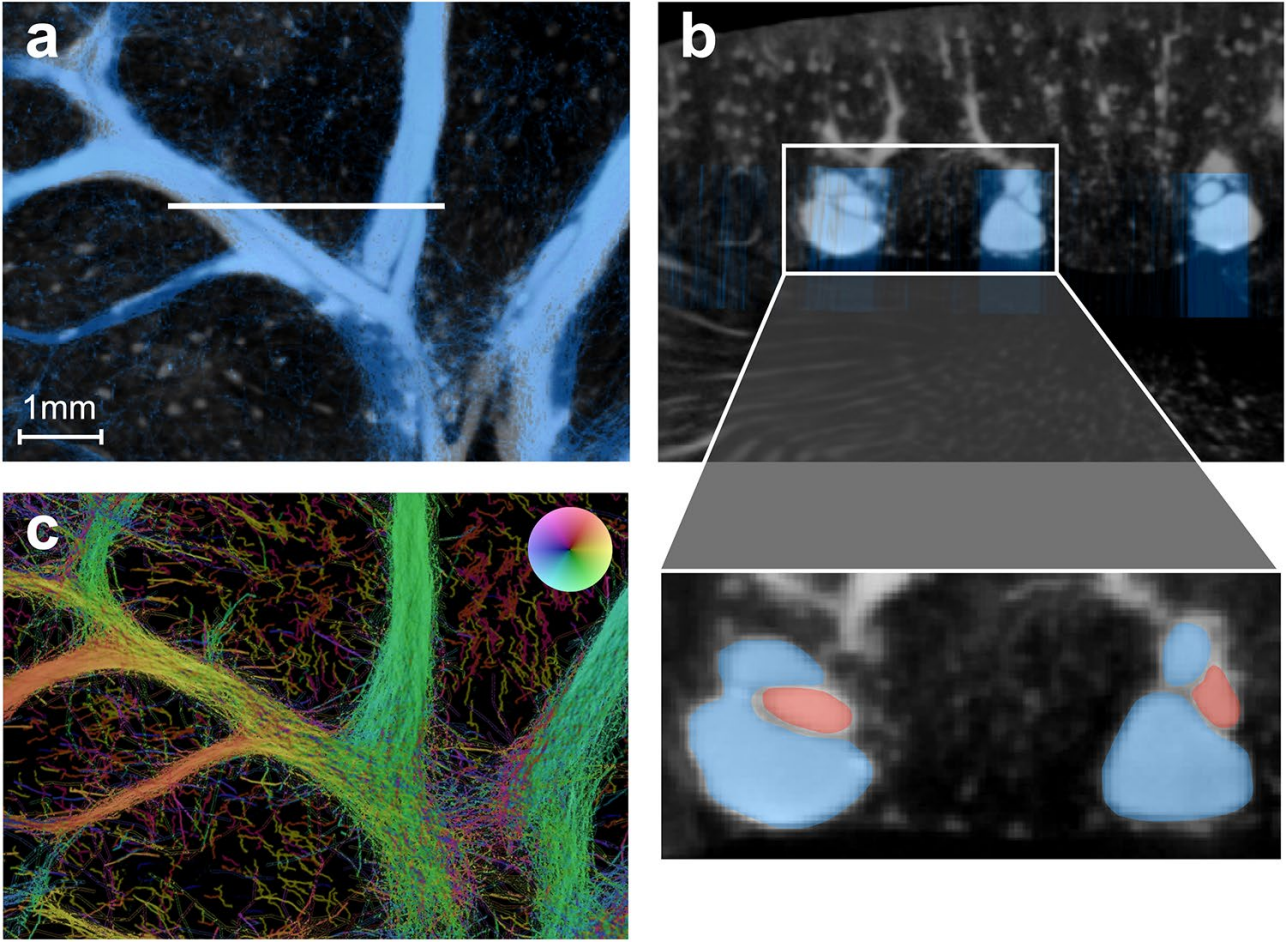
Veins wrapped around their paired artery. (**a**) Section of a coronal image slice of the *μ*CT with overlap of super-resolution ultrasound scan 2 (blue). (**b**) Section of a sagittal image slice of the *μ*CT with overlap of super-resolution ultrasound scan 2 (blue). The up-scaled content shows how the large segmental veins (blue) wrap around their paired segmental artery (red). (**c**) Unfiltered microbubble track map of the same slice as (**a**) (for visualization, the color transparency in the microbubble track map is scaled by the intensity map). The arteries are not readily apparent in the microbubble track maps without filtering and separating the microbubbles with artery and vein flow direction, as shown in Fig. 3b-2.

The segmental and larger arcuate arteries displayed in the SRUS image in Fig. 3b-2 seemed wider than those in the *μ*CT ROI in Fig. 3b-1. The *μ*CT demonstrated how these arteries were partially wrapped by their counterpart vein (Fig. 4a,b). Not only are the MBs in the arteries difficult to track due to a pulsating flow with high peak-systolic velocities, but the close proximity with the veins makes it even more challenging; on the unfiltered MB track maps, the artery tracks seemed hidden in the large number of vein tracks surrounding them, as exemplified in Fig. 4c. The MB track map of scan 2 included 4460 segmental/large arcuate artery tracks versus 12,300 segmental/large arcuate vein tracks (number of MB links associated with the arteries: 27,000 vs. number of MB links associated with veins: 83,230). Additionally, the length of tracks associated with the arteries was shorter than the length of tracks associated with the veins (median length of artery tracks: 457 μm vs. median length of vein tracks: 739 μm), indicating that it is difficult to link the fast-flowing arterial MBs from frame to frame.

**Figure 5:**
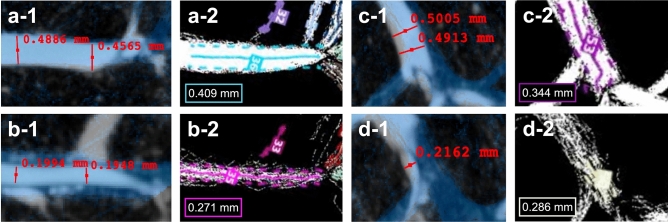
Examples of vessel diameter measurements. (**a-1**) Arcuate vein in the *μ*CT with blue super-resolution ultrasound image overlap, shows diameter measurements of 0.489 mm and 0.457 mm. (**a-2**) Corresponding vessel in the microbubble track map showing a mean diameter, estimated around the turquoise centerline, of 0.409 mm. (**b-1**) shows the arcuate artery paired with (**a-1**) with diameter measurements of 0.199 mm and 0.195 mm. (**b-2**) Corresponding vessel in the microbubble track map showing a mean diameter estimation of 0.271 mm. (**c-1**) Another example of an arcuate vein in the *μ*CT with diameter measurements of 0.501 mm and 0.491 mm. (**c-2**) Corresponding microbubble track map with a mean diameter estimation of 0.344 mm. (**d-1**) Paired arcuate artery in the *μ*CT with diameter measurements of 0.216 mm. (**d-2**) Corresponding artery in the microbubble track map with estimated diameter of 0.286 mm.

Examples of diameter measurements from two larger, paired arcuate veins and arteries are shown in Fig. 5. The measurements showed a tendency to underestimate the vein diameter and overestimate the artery diameter in the SRUS images relative to the *μ*CT, when *4 s.d*. (standard deviation) of the MB position around the centerline was considered the diameter measure in the SRUS data.

**Figure 6:**
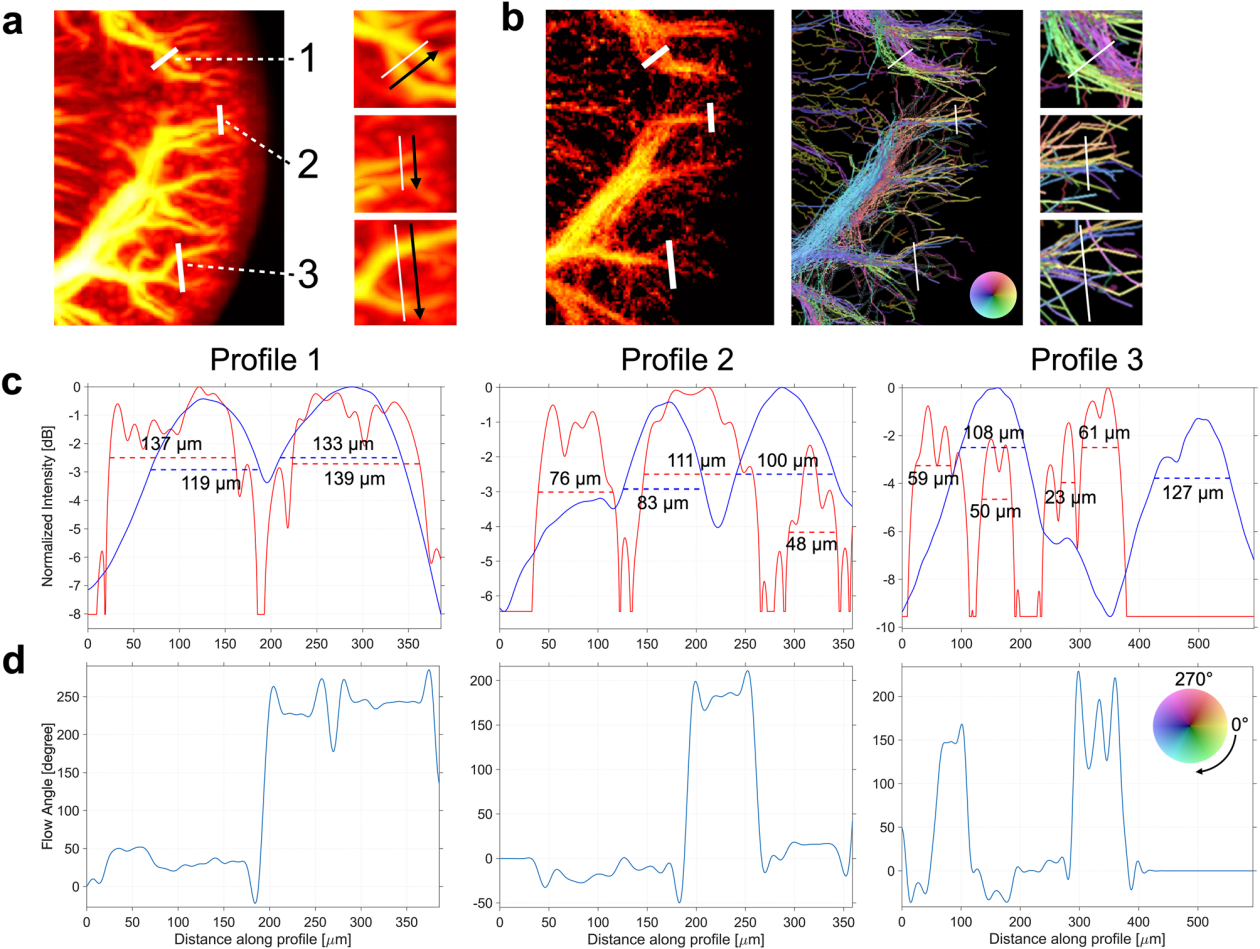
Line intensity profile examples. (**a**) *μ*CT maximum intensity projection (MIP) with three lines for profile intensity placed across different vessels. The arrows indicate the direction of the profiles. (**b**) Corresponding super-resolution ultrasound intensity map (left) and microbubble track map (right). For visualization, the color transparency in the microbubble track map is scaled by the intensity map; this scaling is not applied in the zoomed-in boxes and for the intensity profiles. (**c**) Shows the three intensity profiles. The blue line is the *μ*CT MIP intensity profile, the red line is the super-resolution ultrasound intensity profile. The super-resolution ultrasound intensity profile is scaled according to the *μ*CT intensity. The size shows the -3 dB width of the vessels. (**d**) Shows the angle of the flow for the super-resolution ultrasound image profiles. This reveals opposite flow directions, separating artery and vein tracks.

The cortical radial arteries with diameters of ~ 50 μm were found as the limit of structures that could be identified with the *μ*CT resolution used in our study. At this level, comparison becomes challenging, and discrepancies will arise. To illustrate the challenges with direct comparison of these vessels, three sampled line intensity profiles are shown in Fig. 6. In profile 1, two vessels intertwined in the *μ*CT MIP (Fig. 6a). When comparing the intensity profiles from the *μ*CT MIP and SRUS image in Fig. 6c, there seemed to be two vessels with similar size and location in both images. Judging by the direction coloring in Fig. 6b (right) and the flow angles in Fig. 6d, one of the vessels was an artery and the other was a vein. However, the two vessels on the *μ*CT MIP were both vein branches; this was revealed when inspecting the *μ*CT volume. The artery or arteries running along these veins were too small to visualize in the *μ*CT properly but were caught in the ultrasound SRUS image. The size of the artery suggests an overestimation of the artery dimensions in the SRUS images, as was also seen in Fig. 5. Additionally, two separate veins were not readily visible from the SRUS image. This can occur because the veins were closer than the MB localization uncertainty. Profiles 2 and 3 show smaller vessels. The *μ*CT intensity profile 2 shows two veins. The corresponding SRUS image profile indicates three arteries and only one vein crossing that same profile line. Again, the veins were not individually resolved in the SRUS image. In addition, it was not possible to verify whether the artery MB tracks represented one or more arteries or whether some were false tracks, again due to the *μ*CT voxel size. For profile 3, the two veins were spatially further apart and displayed in both intensity profiles, along with arteries in the SRUS intensity profile. Lastly, profiles 2 and 3 also revealed how the global transform-based co-registration led to vessels that did not overlap completely; both *μ*CT intensity profiles are shifted slightly right to the corresponding SRUS image intensity profiles.

**Figure 7:**
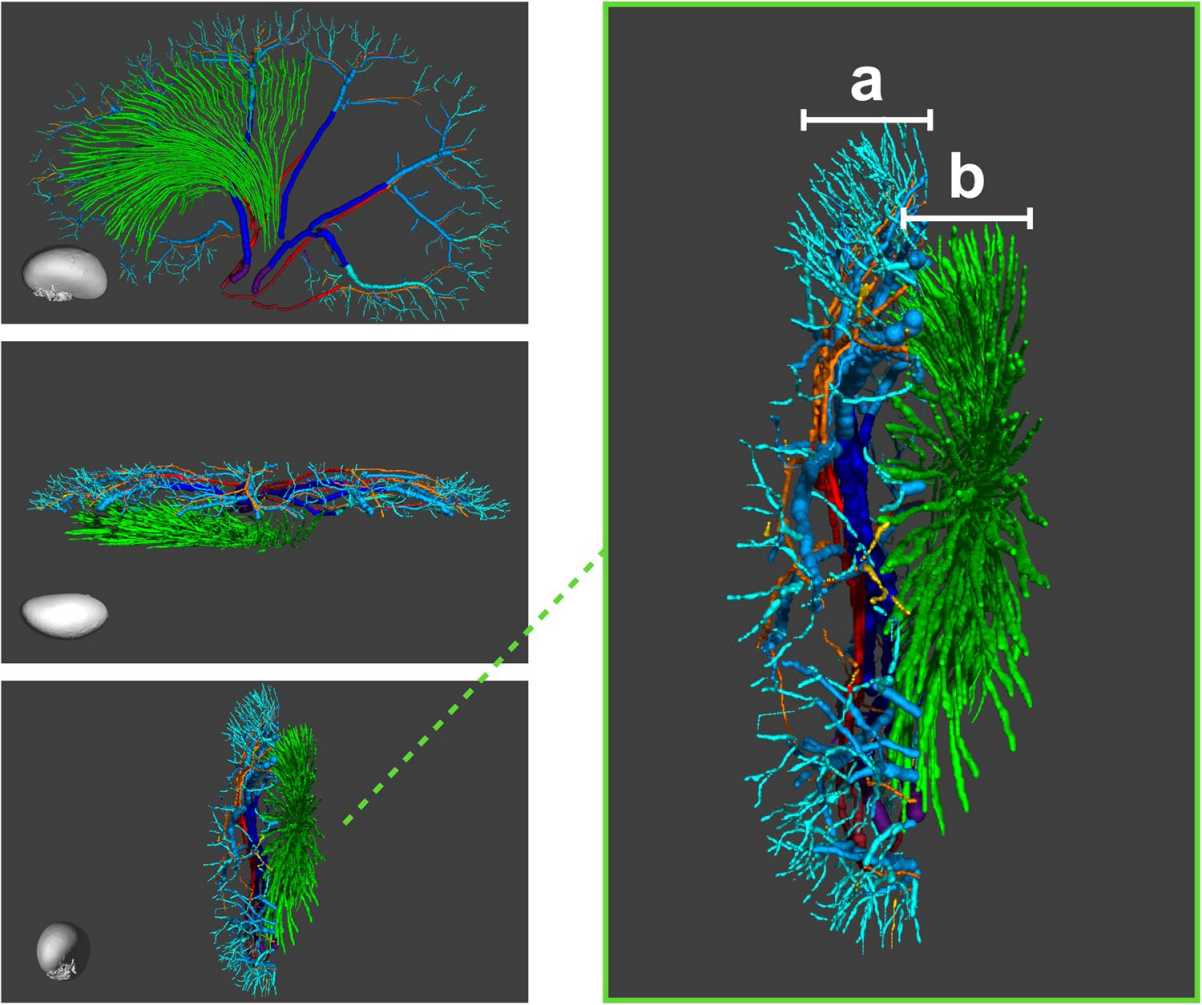
3D illustration with rotation of the manually drawn vessel centerlines in the *μ*CT. The 3D illustration shows the vasa recta bundles (green, descending and ascending vasa recta are not distinguishable), and larger arteries and veins (purple = renal vein branches, dark blue = segmental veins, blue = arcuate veins, turquoise = cortical radial veins, dark red = renal artery branches, red = segmental arteries, orange = arcuate arteries, yellow = cortical radial arteries). Insert: (**a**) shows the *μ*CT vessel centerlines in super-resolution ultrasound scan 2 overlap. (**b**) shows approximately half of the well-resolved vasa recta segmented in the *μ*CT in the super-resolution ultrasound scan 1 overlap.

A rotation of the *μ*CT vessel centerlines revealed the complexity of the 3D vessel structure captured in the 2D SRUS images; Fig. 7 illustrates how multiple vessels lay displaced from each other, winding in the elevational plane of the ultrasound beam, putting further emphasis on the challenge of tracking intravascular MBs in a 2D imaging space.

Figure 7 also demonstrates that not only are the cortical structures difficult to capture in 2D, but vessels that are uniform and placed in parallel, such as the vasa recta, pose certain challenges too. Within the SRUS image and *μ*CT overlap of scan 1, we found up to nine individual superposed vasa recta bundles in the *μ*CT that could reasonably contribute with a microbubble track to a single SRUS image pixel (Supplementary Fig. S3). Therefore, the vasa recta depicted in the SRUS images were likely a summation from a couple or more different vasa recta bundles.

## Discussion

SRUS has been developed for in vivo studies of both the healthy and diseased microvasculature and shows a great clinical potential^3,9,10,22,23,30,31^. The technique offers a fast investigation of the microvascular architecture, gives quantitative MB velocity estimates as a surrogate measure for microvascular blood flow velocity, and is adaptable to humans. However, due to the high complexity of the microvascular systems^32^, comparing the structures depicted in the microbubble-based SRUS images with other microvessel imaging modalities and showing their similarities would further support the technique. In this study, we compared two in vivo 2D SRUS images of a rat kidney with the corresponding MIPs from an ex vivo *μ*CT scan of the same kidney. The two imaging modalities showed visually very similar vascular features, illustrating a high vessel branching complexity captured in the 2D SRUS images. The results also illustrated critical challenges in validating SRUS with ex vivo *μ*CT at the given resolution quantitatively. In this feasibility study, the presented vessel overlap percentages represent a natural initial attempt. In order to appreciate these scores and their uncertainty, it is important to note the many factors that influence them. Firstly, the comparison included primarily the segmental and larger arcuate vessels, i.e*.*, vessels ranging from ~ 100 to 1300 μm in diameter. Validating vessels below our ultrasound system's diffraction limit (half the ultrasound wavelength) would mean validating small vessels spatially closer than ~ 125 μm, which we did not achieve, primarily due to the insufficient resolution of the *μ*CT used in this study. Secondly, only a single specimen was included. Including more specimens in a larger-scaled study is required to estimate the statistical variation in the overlap measures. Shifting the *μ*CT ROIs used for comparison could provide some insight into how sensitive the overlap measures are to proper co-registration. However, without a procedure to do this systematically, i.e*.*, having a complete annotation of all vessels in the *μ*CT, deciding the right kind of ROI displacement type and length is challenging, and it would be hard to draw any conclusions from such an analysis. Therefore, the *μ*CT ROI mirroring was our only feasible option for this initial study. Lastly, manual labeling is time-consuming and not suited for neither larger-scaled studies nor whole specimen vessel annotation. The lack of automatic approaches for evaluating microvascular network organizations is a bottleneck for taking the SRUS validation to the next level^33,34^.

For future studies, validating microvascular structures such as afferent arterioles or single vasa recta within the vessel bundles will require optimizing both the SRUS images and the *μ*CT resolution. The low contrast-to-noise ratio in our SRUS data resulted in some disorganized trajectories in the unfiltered SRUS images, especially in the renal cortex, as evident from Fig. 1b-1,c-1. Given that the SRUS images were obtained in vivo with a modified but commercial ultrasound scanner, this is still a substantial improvement compared with previously available techniques for rodent kidney imaging. However, a higher contrast-to-noise ratio is needed to detect and track the MBs more accurately to improve the image quality for the smaller vessels in the cortex. Since the renal cortex has a high blood flow compared with the medulla, improvements in MB tracking in the cortex could be obtained by, e.g*.*, lowering the MB concentration further or increasing the transmission frequency^11,12^. Related to the *μ*CT, we demonstrated the challenging task of capturing the vasculature in a whole organ, as image resolution and field of view are inversely correlated. We chose to include the entire kidney in the *μ*CT scan to match the SRUS images. Image comparisons can be made at a 5–10 μm scale using a smaller isometric voxel size in a selected smaller region of the kidney^24,26^. In Zhu et al.^21^, they used a 4-μm voxel image to compare vessel size distribution in whole rabbit lymph nodes, which is possible because the lymph nodes are much smaller than the rat kidneys. However, even at this level, the renal cortical arteries and arterioles can still be difficult to dissolve given their small size and often close parallel run with the large veins, as exemplified by Nordsletten et al., where a 4-μm voxel image was inadequate for resolving all the cortical arteries and arterioles^35^. Nonetheless, this possibility should still be investigated in future research, and new synchrotron imaging will enable the necessary high resolution in a large volume^36^. We labeled the vessels in the *μ*CT in an antegrade manner, starting from the segmental arteries and veins. A commonly used method for organizing the structural information in the vascular networks is the Strahler Ordering, where the vessels are labeled retrogradely, as the resulting vessel categories have shown to correlate well with the vessel radii^35,37,38^. For our study, the antegrade labeling was considered sufficient, but for future studies with a more detailed categorization of the renal vascular tree, i.e*.*, how the cortical radial arteries branch, a Strahler approach should be considered.

The percentage of image overlap was higher for the veins than the arteries, which a number of factors can explain: Firstly, the veins are voluminous compared with the arteries, and the number of veins segments was six times higher than artery segments. As the *μ*CT ROIs represent only the well-resolved vessels, the arterial tree was not fully appreciated^35^. Secondly, the images were manually co-registered with a global similarity transformation. Due to tissue deformation during ex vivo specimen preparation, and the fact that the thick SRUS image slices had to fit across roughly 80 thinner *μ*CT image slices, the overlap was not perfect, as exemplified in Fig. 5c-1. Even a small incongruence in the image registration affects the vessel overlap percentage, although the same vessels are there. Lastly, we compared two fundamentally different imaging modalities. Each of the modalities attained the 2D-projected representations of the 3D vascular structures differently, which inevitably will lead to incomplete vessel overlap^39^.

We also compared vessel diameter measurements from two larger, paired arcuate arteries and veins, as shown in Fig. 5. These examples pointed toward underestimating the SRUS-derived vein diameters and overestimating the artery diameters compared with those from the *μ*CT. However, vessel diameters from both imaging modalities are likely to diverge from the actual in vivo vessel diameters. For the *μ*CT images, the ex vivo specimen preparation changes vessel proportions due to removing the effect of neural or chemical signals that cause either vessel constriction or dilation. Additional factors such as tissue swelling, perfusion pressure during contrast administration, and the effect of contrast curing and paraffin embedding will affect the vessel dimensions^40,41^. Moreover, a smaller isometric voxel size is necessary for a more precise vessel delimitation. The dilated vessel centerlines from the *μ*CT were not used for vessel size estimation but only for creating ROIs in which we expected to find MB tracks. A more systematic direct comparison of specific vessel diameters using *μ*CT is a more elaborate procedure that would require corresponding branching points and vessel segments to be identified: an arduous task when working with 2D SRUS, where individual vessels overlap. For the SRUS images, when the MB localization is subject to uncertainty, so are the SRUS-derived vessel diameters. Additionally, the diameter of a given vessel relies on the number of MBs passing through^11–14^. For simplicity, we used *4 s.d*. as a measure for the diameter. Other approaches could be investigated in future studies. In a recent SRUS study, the Euclidean distance from a vessel centerline to the nearest point on the vessel border was used^20^. The vessel diameter is an essential physiological metric. Using diameters to calculate blood flow metrics such as vascular resistance requires meticulous and accurate measurements, as even minor variations in diameter substantially affect the results. As for now, the diameter measurements from the SRUS images do not seem useful in examining acute and delicate vessel caliber changes but have the potential to give relevant information when examining chronic diseases with vascular alterations together with metrics such as vessel density, branching, or tortuosity^3,9,10,25,32^. Finally, even though different 2D approaches have been used to examine microvascular alterations^3,9,10,42,43^, and can give meaningful insight into disease progression and treatment responses, only 3D imaging will truly grasp the pathological alterations that occur in the microvascular anatomy^25,44^. 3D acquisition of SRUS data will also aid MB tracking by accounting for the elevational dimension of the MB flow^15,20,21,45,46^. For 2D SRUS used on a microvascular disease model, a great advantage of using 3D *μ*CT for comparison is the possibility to confirm any 2D-derived pathological microvascular parameters^32^.

Even if we succeeded with validating vessels below the diffraction limit, these might still be relatively large vessels, such as the cortical radial arteries; however, the medullary and cortical microvasculature comprise the clinically interesting areas of the kidney. The renal microvessels are all in size range of 20 μm and smaller, and they are densely packed in a complex 3D network, making them very difficult to isolate with SRUS. Our results did show an apparent visual similarity in the vascular patterns of the medullary vasa recta between the two imaging modalities, and SRUS allowed differentiation between the descending and ascending vessels. However, we could not quantify their similarity as described in the “Results” section. The vasa recta are 20-μm vessels organized in vascular bundles, and they are clinically interesting, as they are central for diluting and concentrating urine. Further, they supply the part of the kidney most vulnerable to ischemic damage: the outer medulla. Thus, they are central in the development of acute and chronic renal failure^47–49^. Medullary oxygen deficiency is also suspected to be part of diseases predisposing to renal failure, e.g*.*, diabetes and hypertension. All things considered, further knowledge on medullary perfusion is clinically desirable. It was also clear from the results that tracking MBs from complex 3D vessel networks in 2D is challenging. Studies on optimizing MB tracking to best link the MBs in sequential image frames have been essential for improving the final SRUS image^19,50–52^. Depending on the elevation depth of the ultrasound beam, the SRUS images will include multiple vessels winding and lying displaced in front of each other in the elevational plane, as shown in Fig. 7. Therefore, when the MB contrast data are processed in a 2D image space, MB trajectories that appear as one vessel can represent a sum of several overlapping vessels, as demonstrated with the vasa recta in Supplementary Fig. S3, or two neighboring vessels, as demonstrated in Fig. 6. Additionally, MBs that traverse in overlapping vessels can be wrongfully linked as a non-existing vessel, especially in areas with dense vasculature and a high MB concentration. Consequently, the challenge in the future is how to grasp the complexity of closely related and intertwined microvessels, such as those in the renal cortical network with the afferent and efferent arterioles entering and exiting the glomerulus and the surrounding densely masked peritubular capillary network^24,53^. The microvasculature of the renal cortex is—like the vasa recta—essential in the maintenance of various kidney functions, e.g*.*, the afferent and efferent arterioles affect the hydrostatic pressure within the glomerular capillaries and thereby the glomerular filtration; the production of renin from the afferent arterioles helps regulate the arterial blood pressure; and the peritubular capillaries that travel along the renal tubules allow absorption of water and solutes and secretion of organic solutes together with the vasa recta. These cortical vascular components are affected in a range of renal diseases, e.g*.*, diabetic nephropathy^7,8,54^, hypertensive nepropathy^55^, and arteriosclerosis^56^, ultimately decreasing renal function.

In conclusion, this feasibility study showed that in vivo SRUS images correspond visually very well with ex vivo *μ*CT of the same rat kidney. It was also shown that it is challenging to use *μ*CT data at the chosen resolution to validate the super-resolution capability of ultrasound, i.e*.*, validating vessels in the actual microvasculature of the kidneys. Critical challenges in 2D SRUS were identified, e.g*.*, the complexity of projecting a 3D vessel network into 2D; these challenges should be considered when interpreting clinical or preclinical SRUS data in future studies.

## Materials and methods

### Ethical considerations

The reporting in this manuscript follows the recommendations in the ARRIVE guidelines. The experiment was conducted in agreement with approved protocols (approval granted from the Danish Animal Experiments Inspectorate under the Ministry of Environment and Food, Denmark). The SRUS scans and *μ*CT specimen preparation were performed at the University of Copenhagen, and all procedures agreed with the ethical standard of the university, which meets that of the EU Directive 2010/63/EU for animal experiments. The study was conducted on a healthy male Sprague–Dawley rat (weight: 330 g. Janvier Labs, Le Genest-Saint-Isle, France). The rat was housed at the university’s animal facility at the Department of Experimental Medicine, where animal caretakers were responsible for its wellbeing. The rat was housed in the company of another rat and held in a 12/12-h light/dark cycle with standard chow and water freely accessible.

### In vivo super-resolution ultrasound imaging

The rat was scanned during laparotomy. The rat was anesthetized in a small chamber with 5% isoflurane delivered in 65% nitrogen and 35% oxygen. Ventilation was secured through a tracheostomy tube connected to a mechanical ventilator (Ugo Basile, Gemonio, Italy) with 69 respirations/min. A 2% isoflurane concentration upheld the anesthesia. The left jugular vein was catheterized with two polyethylene catheters (PE-10) for infusion of ultrasound contrast (SonoVue, Bracco Imaging, Milan, Italy) and isotonic saline with the muscle relaxant Nimbex (cisatracurium, 0.85 mg/ml, GlaxoSmithKline, London, United Kingdom, 20 μl/min). A polyethylene catheter (PE-50) in the left carotid artery and a Statham P23-dB pressure transducer (Gould, Oxnard, CA, USA) ensured continuous monitoring of the mean arterial pressure (MAP). The rat was placed in the supine position on a heating table to ensure a steady body temperature (37 °C). Laparotomy exposed the left kidney, and a metal retractor kept the left side of the diaphragm, ventricle, and spleen away from the kidney. The rat was scanned with a BK5000 scanner and an X18L5s hockey-stick transducer (BK Medical ApS, Herlev, Denmark). The scanner was modified to allow live streaming of beamformed radio-frequency data to a disk. Two different coronal imaging planes were found with B-mode image guidance: One imaging plane in the renal center showing the medulla’s vasa recta (scan 1), and one imaging plane more ventrally showing the segmental and larger arcuate arteries and veins (scan 2). After adjustment of the image plane, the transducer was fixated with a stand. A pump infused the 1:15 dilution of MBs in isotonic saline at 100 μl/min. The infusion was adjusted to have isolated MBs for localization and tracking (a video of the microbubble signal from SRUS scan 1 can be seen in Supplementary Video S4). Data acquisition started when the MBs reached the renal vasculature. Each SRUS scan was acquired over 10 min. Data were acquired with line-per-line focused beam transmission (frame rate: 54 Hz, center frequency for transmission: 6 MHz, mechanical index: 0.2). An amplitude modulation sequence (half-power, full-power, half-power) generated the contrast images, and interleaved B-mode images were used for motion compensation. Non-rigid motion was estimated with speckle tracking in the renal tissue. The motion was compensated using the displacement estimates to adjust each MB back to its location on a reference image^57,58^. MBs were localized using thresholding and centroid detection, and MB trajectories were made using a modified Kalman tracker^52^ (maximum linking distance: 278 μm. Only trajectories of MBs that were observed in least three consecutive frames were considered a track). Afterward, the trajectories were inserted in high-resolution images to generate the SRUS images. The MB direction was displayed in color-coded MB track maps with a color wheel indicating the direction for a distinction between arterial and venous flow. Brighter colors correspond to faster MB velocities.

### Ex vivo μCT imaging

After SRUS, ligatures were prepared around the left renal artery, renal vein, and ureter as well as the aorta both in the caudal part for catheter fixation and above the left renal artery but below the right renal artery. The rat was heparinized (Heparin “SAD” 1000 IE/ml, Amgros, Copenhagen, Denmark) with 1000 IE/kg intravenously. Once heparinized, the abdominal aorta was catheterized with a PE-50 catheter with the catheter tip placed at the left renal artery, and the ligature around the aorta above the left renal artery was tightened. A ligature near the inferior vena cava occluded the left renal vein, and a small hole cut in the vein allowed the injection media to leave the renal vasculature. The left kidney was perfused with 8 ml heparinized saline (1000 IE/ml heparin diluted 1:100 in isotonic saline) at 2 ml/min. The heparin solution was pre-heated to 40 °C. The flushing continued until only clear saline ran from the renal vein. During the renal vascular flushing, the rat was euthanized by decapitation. Directly after flushing, 3 ml of μAngiofil contrast agent and hardener mixed according to the manufacturer’s guidelines (Fumedica AG, Muri, Switzerland) was infused at 1 ml/min^26,40^. The infusion continued until the entire surface of the kidney was blue, and a considerable amount of contrast had left the renal vein. The kidney was left for 30 min, allowing contrast hardening. Afterward, the kidney was excised, decapsulated and fixated in formaldehyde, followed by embedment in paraffin in a custom-made cylinder-shaped holder. The kidney was scanned for 11 h in a ZEISS XRadia 410 Versa *μ*CT scanner (Carl Zeiss Microscopy GmbH, Jena, Germany) at the following settings: isotropic voxel size 22.6 μm, 50 kV tube voltage, 0.2 mA current, appertaining LE3 filter, 360° scan around the vertical axis with 3201 different projections (0.112° rotation steps).

### Image co-registration and analysis

To obtain images from the *μ*CT volume with the same vessels as the SRUS images, we made MIPs of the *μ*CT slices approximately covering the volume depicted in the SRUS images. Prior to co-registration, the 2D SRUS images were assigned a constant elevation depth of 1.8 mm, such that each SRUS image was approximated as a rectangular field-of-view of 15.3 × 21.5 × 1.8 mm. The SRUS scans were manually co-registered to the *μ*CT volume using ITK-SNAP (version 3.8.0)^59^. Due to tissue deformation during *μ*CT specimen preparation, a local non-rigid registration would be ideal. Differences in resolutions, fields of view, contrast mechanisms and the 2D vs 3D nature of the problem would make this a challenging task, requiring a detailed study on its own. Restricted to a global transformation, we allowed for translation, rotation and scaling to achieve a visually satisfying agreement. From this, we calculated the MIPs of the *μ*CT within the SRUS overlaps (in the direction of the elevation plane). Afterward, vessel centerlines were manually drawn and labeled in the *μ*CT in the SRUS image overlaps. The included vessels were the visible, contrast-filled segmental, arcuate and cortical radial arteries and veins found in the SRUS image overlap of scan 2, and the cranial half of the vasa recta visible in the overlap of SRUS scan 1^60^. Each centerline was found manually using the three different imaging planes (coronal, sagittal, and axial). Depending on the orientation of the vessel, the plane where the vessel appeared most circular (in cross section) was used to find the approximate center. The *μ*CT centerlines were drawn slice by slice starting from the renal artery and vein branches in the hilum in an antegrade manner ending in the cortical radial vessels. The vessels were separated into arteries and veins at this level, based on their size: the veins have a substantially larger diameter than their paired artery (illustrated in Fig. 4b). All the centerlines from the 3D *μ*CT were projected into the coordinate system of the 2D SRUS images using the inverse similarity transform from the co-registration. To cover the contrast-filled areas, the transformed *μ*CT centerlines were dilated based on approximate expected vessel diameters. The expected diameters were extracted from selected examples of diameter measurements of the segmental, arcuate, and cortical radial arteries and veins at different branching levels on the *μ*CT. From this, one ROI with all the arteries and one ROI with all the veins were created for the vessel overlap estimations.

On the SRUS scans, different regions were marked and labeled into either “segmental or larger arcuate vessels”, “cortex” (including all vessels that were located superficially to the larger arcuate vessels that traverse on the border between cortex and medulla; hence, this region could include smaller branches of arcuate vessels, the cortical radial vessels, and possibly also tracks from the renal microvasculature), “outer medulla”, or “inner medulla” using *MATLAB* (Math Works, Inc., version R2020b). Each label consisted of several smaller regions in which the arterial flow direction was determined, which allowed separation of artery and vein MB trajectories. An example of these regions can be seen in Supplementary Fig. S5. In scan 2, the vessel centerlines of well-defined MB trajectories in the filtered MB track maps of the arteries and veins, respectively, were manually plotted.

For scan 2, we compared vessel overlap: Firstly, the percentage of vessel centerlines from the MB track maps that were recovered within the *μ*CT vessel ROIs was calculated separately for the veins and arteries. Secondly, because the well-defined bundles of MB trajectories in the MB track maps that were used to manually draw the vessel centerlines represented primarily larger vessel, thereby excluding the smaller cortical MB tracks, the percentage of the area with MB tracks (all non-zero pixels in the filtered track map) covered by the *μ*CT vessel ROIs was also calculated. Additionally, examples of vessel diameters were compared. In the *μ*CT scan, the diameters were manually measured in ITK-SNAP. For the SRUS images, based on the assumption that vessels have a tubular structure with a Gaussian profile, a simplistic measure for vessel diameter was considered as *4 s.d*. of the MB’s positions around the centerline for the specific vessel. These centerlines were automatically extracted based on the skeleton of the artery and vein track maps in each of the labeled regions.

### Equipment and settings

All the SRUS images in Figs. 1, 2, 3, 4, 5, and 6, the *μ*CT images in Figs. 1, 2, 3, and 6 (including the *μ*CT volumes of the kidney in Fig. 1a), and the supplementary figures and video were created in *MATLAB* (version R2019b and R2020b, Mathworks, U.S.). The *μ*CT images with SRUS image overlap in Figs. 4 and 5 were created in ITK-SNAP (version 3.8.0)^59^. The rendering of the *μ*CT segmentation in Fig. 7 was done with Blender (version 2.91, www.blender.org). All the figures were gathered, set up, and annotated in Keynote (version 10.3.8, Apple Inc).

## Supplementary Information

Supplementary Information 1.

Supplementary Video 1.

## Data Availability

Raw data and image processing algorithms can be exchanged through a collaboration agreement. Processed data and analysis algorithms can be made available upon request.
