# Supplementary material for: Evaluation of 2D super-resolution ultrasound imaging of the rat renal vasculature using ex vivo micro-computed tomography
Source: Sci Rep. 2021 Dec 21;11:24335. doi: 10.1038/s41598-021-03726-6 (PMC8692475; doi:10.1038/s41598-021-03726-6)
Supplement: Supplementary file 1 — Supplementary Information 1. [file 41598_2021_3726_MOESM1_ESM.pdf]

Supplementary Information

Evaluation of 2D Super-Resolution Ultrasound Imaging of the Rat Renal Vasculature using Ex Vivo Micro-Computed Tomography

Sofie Bech Andersen[1,2*], Iman Taghavi[3], Hans Martin Kjer[4], Stinne Byrholdt Søgaard[1,2], Carsten Gundlach[5], Vedrana Andersen Dahl[4], Michael Bachmann Nielsen[2,6], Anders Bjorholm Dahl[4], Jørgen Arendt Jensen[3], Charlotte Mehlin Sørensen[1].

[1] Department of Biomedical Sciences, University of Copenhagen, 2200 Copenhagen, Denmark
[2] Department of Radiology, Rigshospitalet, 2100 Copenhagen, Denmark
[3] Center for Fast Ultrasound Imaging, Department of Health Technology, Technical University of Denmark, 2800 Lyngby, Denmark
[4] Department of Applied Mathematics and Computer Science, Technical University of Denmark, 2800 Lyngby, Denmark
[5] Department of Physics, Technical University of Denmark, 2800 Lyngby, Denmark
[6] Department of Clinical Medicine, University of Copenhagen, 2200 Copenhagen, Denmark
*Corresponding author: anne.sofie.bech.andersen@regionh.dk

Supplementary figures

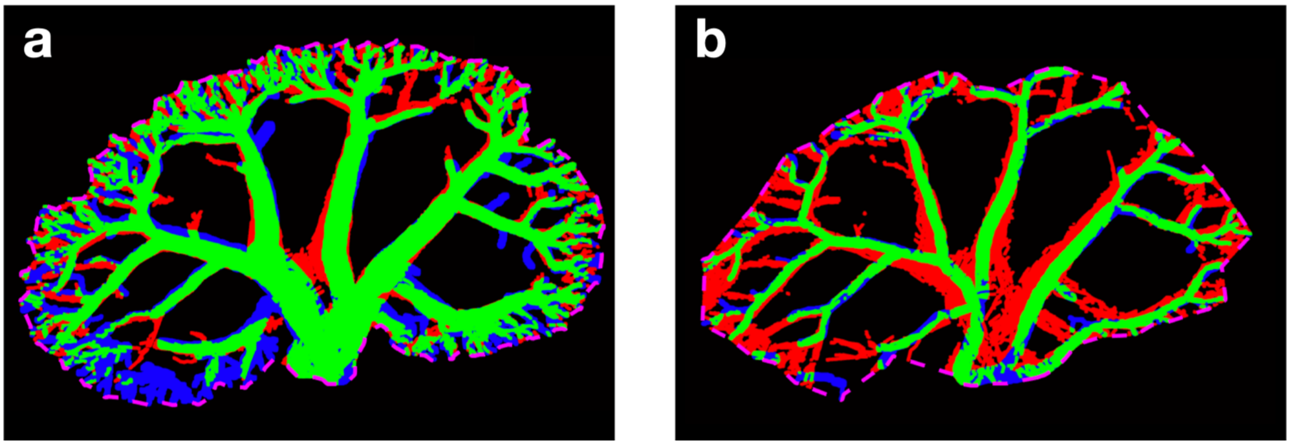

Supplementary Figure S1. Overlap of non-zero pixels from the filtered microbubble track maps and the μCT ROIs of scan 2. **a)** Vein overlap (percentage of non-zero pixels recovered in the μCT ROI = 77.4%). **b)** Artery overlap (percentage of non-zero pixels recovered in the μCT ROI = 44.3%). Blue = μCT ROI. Green = overlap of microbubble track map and μCT ROI. Red = microbubble track map non-zero pixels outside the μCT ROI.

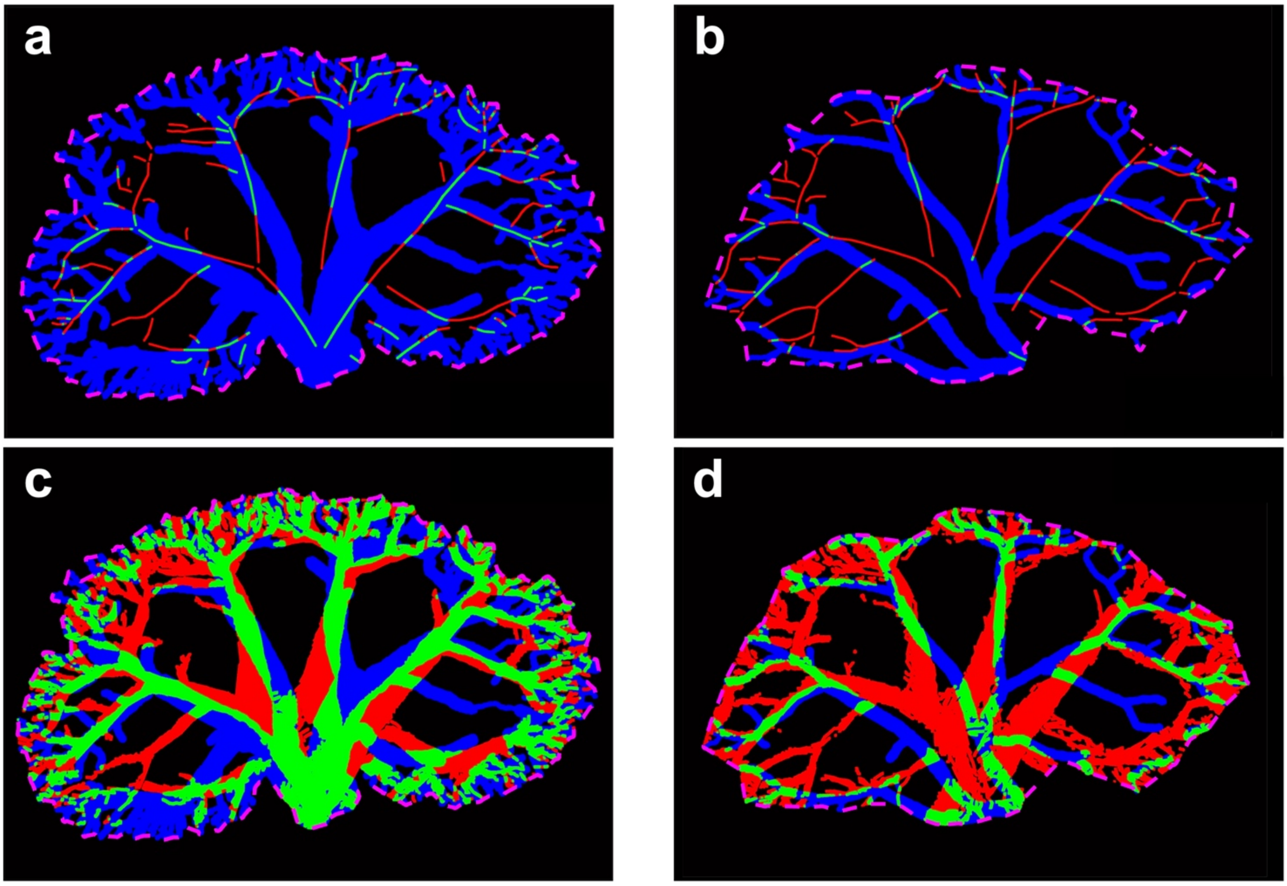

Supplementary Figure S2. Vessel overlap control.

As a reference for the vessel overlap percentages in Fig. 3 and Supplementary Fig. S1, we mirrored the μCT ROIs along the central axial axis and calculated vessel overlap again. This was considered likely to represent a best-case scenario for spurious overlap.

a) and b) show overlap of the manually drawn vessel centerlines in the super-resolution image (green and red lines) of the vein tracks (**a**) and artery tracks (**b**) and mirrored μCT vessel ROIs (blue) inside the μCT ROI region (pink dashed line). The green centerlines are overlapping the μCT ROIs, while the red centerlines are not. The overlap dropped from 85% to 50% for the veins and from 65% to 24% for the arteries.

c) and d) show overlap non-zero pixels from the filtered microbubble track maps and mirrored μCT vessel ROIs for veins and arteries, respectively. The percentage of non-zero pixels from the super-resolution images recovered in the μCT ROI dropped from 77% to 51% for the veins and 44% to 23% for the arteries. Blue = μCT ROI. Green = overlap of microbubble track map and μCT ROI. Red = microbubble track map non-zero pixels outside the μCT ROI.

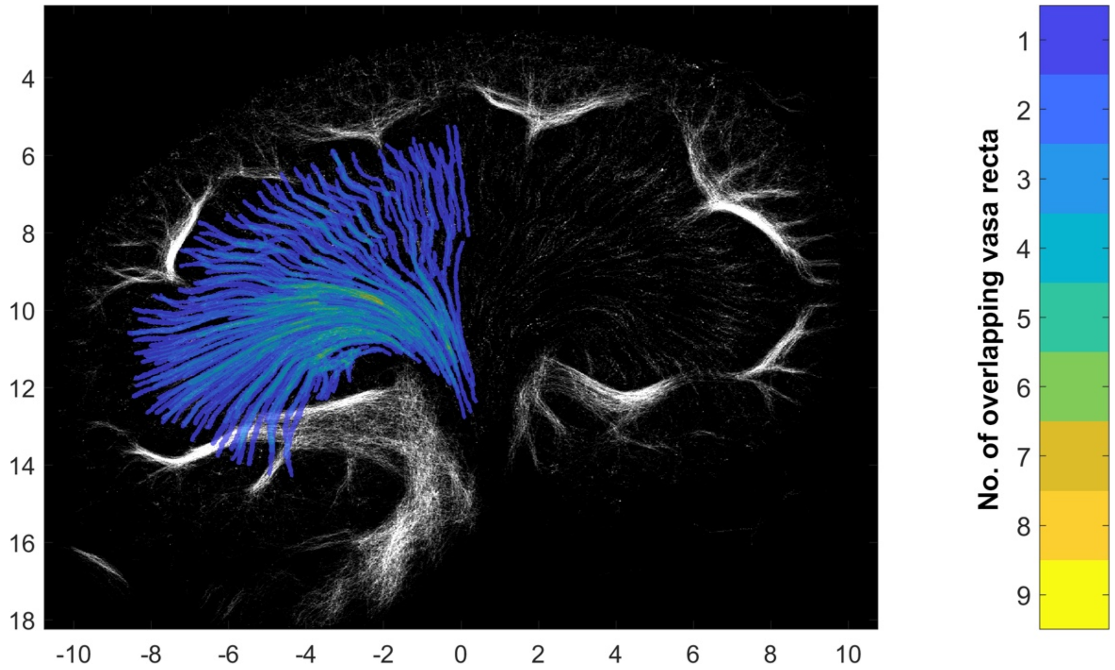

Supplementary Figure S3. μCT vasa recta centerline overlap.
The figure shows the ROI of the vasa recta bundle centerlines annotated in the μCT projected onto ultrasound super-resolution scan 1. The color indicates how many unique vasa recta bundles exist in the μCT and ultrasound super-resolution image overlap. The numbers on the image axes are the ultrasound super-resolution image coordinate system (mm distance).

Supplementary Video S4. Microbubble signal.
The video shows a 19-second example of the microbubble signal from scan 1. The video illustrates how there are more microbubbles in the renal cortex compared with in the medulla's vasa recta due to the high blood flow to the cortex: the cortex receives roughly 90% of the total renal blood flow, while 10% goes to the medulla.

The video is uploaded separately.

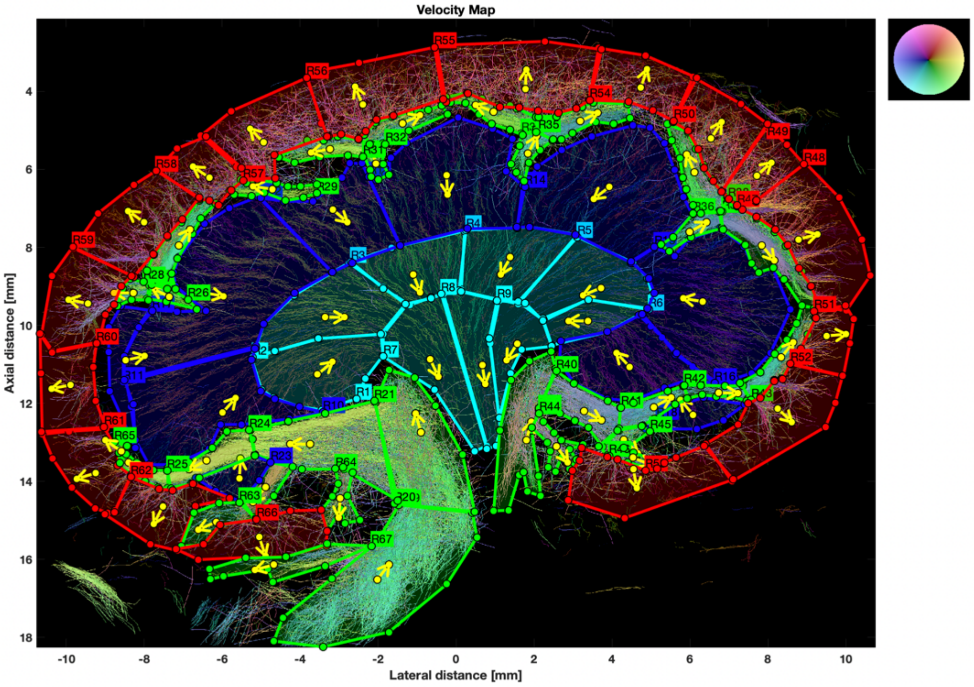

Supplementary Figure S5. Example of regions in the microbubble track map of scan 1.
Green = segmental and larger arcuate vessels, red = cortex, dark blue = outer medulla, turquoise = inner medulla.
The direction of the yellow arrow in each region indicates the arterial flow direction, allowing the separation of artery and vein microbubble tracks in each region.
